# Correction: Suicide and self-harm in low- and middle- income countries during the COVID-19 pandemic: A systematic review

**DOI:** 10.1371/journal.pgph.0006514

**Published:** 2026-05-21

**Authors:** Duleeka Knipe, Ann John, Prianka Padmanathan, Emily Eyles, Dana Dekel, Julian P. T. Higgins, Jason Bantjes, Rakhi Dandona, Catherine Macleod-Hall, Luke A. McGuinness, Lena Schmidt, Roger T. Webb, David Gunnell

In the Funding statement, the grant number from the funder Wellcome Trust is listed incorrectly. The correct grant number is: 204813/Z/16/Z.
