# Supplementary material for: Suicide and self-harm in low- and middle- income countries during the COVID-19 pandemic: A systematic review
Source: PLOS Glob Public Health. 2022 Jun 1;2(6):e0000282. doi: 10.1371/journal.pgph.0000282 (PMC10021274; doi:10.1371/journal.pgph.0000282)
Supplement: S1 Text — (DOCX) [file pgph.0000282.s003.docx]

**Supplementary material**

**Searches used on different databases:**

**Scopus:**

TITLE-ABS-KEY("selfharm*" OR "self harm*" OR "self-harm*" OR "self injur*" OR "selfinjur*" OR "self-injur*" OR "selfmutilat*" OR "self mutilat*" OR "self-mutilat*" OR "suicid*" OR "parasuicid*" OR "suicide" OR "suicidal ideation" OR "attempt* suicide" OR "suicide attempt*" OR "drug overdose" OR "selfpoisoning" OR "self poisoning" OR "self-poisoning" OR "self-injurious behavi*" OR "selfmutilation" OR "self mutilation" OR "self-mutilation" OR "automutilation" OR "suicidal behavi*" OR "selfdestructive behavi*" OR "self destructive behavi*" OR "self-destructive behavi*" OR "selfimmolat*" OR "self-immolat*" OR "self immolat*" OR "cutt*" OR "headbang" OR "head-bang" OR "head bang" OR "overdose" OR "selfinflict*" OR "self-inflict*" OR "self inflict*" OR "hopelessness" OR "powerlessness" OR "helplessness" OR "negative attitude*" OR "emotional negativism" OR "pessimism" OR "depress*" OR "hopelessness depression" OR "passivity" OR "sad-affect" OR "sadness" OR "decreased affect" OR "cognitive rigidity" OR "suicidality" OR "suicide ideation") AND TITLE-ABS-KEY("nCoV" OR "HCoV" OR "covid 19" OR "covid-19" OR "covid19" OR "coronavirus" OR "19 ncov" OR "19-ncov" OR "2019 ncov" OR "2019-ncov" OR "2019ncov" OR "n-cov" OR "ncov" OR "coronavirus disease*" OR "sars-cov-2" OR "sars cov 2" OR "sars-cov 2" OR "mers-cov" OR "mers cov") AND PUBYEAR > 2020

- The filter ‘PUBYEAR > 2020’ corresponds to the 2021 version of this search, in previous years we used ‘PUBYEAR > 2018’ and ‘PUBYEAR > 2019’

**PubMed:**

((mental health[TIAB] OR selfharm*[TIAB] OR self-harm*[TIAB] OR selfinjur*[TIAB] OR self-injur*[TIAB] OR selfmutilat*[TIAB] OR self-mutilat*[TIAB] OR suicid*[TIAB] OR parasuicid*[TIAB) OR (suicide[TIAB] OR suicidal ideation[TIAB] OR attempted suicide[TIAB]) OR (drug overdose[TIAB] OR self?poisoning[TIAB]) OR (self-injurious behavio?r[TIAB] OR self?mutilation[TIAB] OR automutilation[TIAB] OR suicidal behavio?r[TIAB] OR self?destructive behavio?r[TIAB] OR self?immolation[TIAB])) OR (cutt*[TIAB] OR head?bang[TIAB] OR overdose[TIAB] OR self?immolat*[TIAB] OR self?inflict*[TIAB]) OR (hopelessness[TIAB] OR powerlessness[TIAB] OR helplessness[TIAB] OR negative attitude$[TIAB] OR emotional negativism[TIAB] OR pessimism[TIAB] OR depress*[TIAB] OR hopelessness depression[TIAB] OR passivity[TIAB] OR sad-affect[TIAB] OR sadness[TIAB] OR decreased affect[TIAB] OR cognitive rigidity[TIAB] OR suicidality[TIAB] OR suicide ideation[TIAB]))) AND ((coronavirus disease?19[TIAB] OR sars?cov?2[TIAB] OR mers?cov[TIAB]) OR (19?ncov[TIAB] OR 2019?ncov[TIAB] OR n?cov[TIAB]) OR (\"severe acute respiratory syndrome coronavirus 2\" [Supplementary Concept] OR \"COVID-19\" [Supplementary Concept] OR COVID-19 [tw] OR COVID 2019 [tw] OR coronavirus [tw] OR nCoV[TIAB] OR HCoV))

Psy- and SocArXiv (both same query):

"(mental health OR selfharm* OR self-harm* OR selfinjur* OR self-injur* OR selfmutilat* OR self-mutilat* OR suicid* OR parasuicid* OR suicide OR suicidal ideation OR attempted suicide OR drug overdose OR self?poisoning OR self-injurious behavio?r OR self?mutilation OR automutilation OR suicidal behavio?r OR self?destructive behavio?r OR self?immolation OR cutt* OR head?bang OR overdose OR self?immolat* OR self?inflict* OR hopelessness OR powerlessness OR helplessness OR negative attitude OR emotional negativism OR pessimism OR depress* OR hopelessness depression OR passivity OR sad-affect OR sadness OR decreased affect OR cognitive rigidity OR suicidality OR suicide ideation) AND (coronavirus disease?19 OR sars?cov?2 OR mers?cov OR 19?ncov OR 2019?ncov OR n?cov OR COVID-19 OR COVID 2019 OR coronavirus OR nCoV OR HCoV)"

- No date/content filters applied

**Med and BioRxiv, WHO Covid-19 database:**

We directly retrieve ALL new publications related to Covid-19 from these sources, see <http://connect.biorxiv.org/relate/content/181> for Bio- MedRxiv Covid feed and <https://search.bvsalud.org/global-literature-on-novel-coronavirus-2019-ncov/> for WHO on a daily basis. There is no date filter, we retrieve each new report as it becomes available.

We then apply our systematic search to those results, as described here:

*McGuinness et al., (2020). medrxivr: Accessing and searching medRxiv and bioRxiv preprint data in R. Journal of Open Source Software, 5(54), 2651.* [*https://doi.org/10.21105/joss.02651*](https://doi.org/10.21105/joss.02651)

The search strategy is below, please note that the syntax is RegularExpression, but it was designed to correspond to the PubMed query you see above, with new lines being joined with ‘OR’ statements. The only exception is the removal of the AND statement relating to COVID-19 itself, because those 3 sources only include COVID-specific information.

[Ss]elf[- ]?[Ii]njur(y|ious)[- ]?[Bb]ehaviou?r

[Ss]elf[- ]?([Mm]utilat¬[Ii]mmolat)(ion|ed)

[Aa]uto[- ]?[Mm]utilat(ion|ed)

[Ss]uicidal[- ]?[Bb]ehaviou?r

[Ss]elf[- ]?[Dd]estructive)[- ]?[Bb]ehaviou?r

[Ss]uicide

[Aa]ttempted[- ]?[Ss]uicide

[Ss]uicidal[- ]?[Ii]deation

[Ss]elf[- ]?[Hh]arm

[Ss]elf[- ]?[Mm]utilat

[Ss]elf[- ]?[Ii]njur

[Pp]ara[ -]?[Ss]uicid

[Dd]rug[ -]?[Oo]verdose

[Ss]elf[- ]?[Pp]oison(ing|ed)

[Ss]elf[- ]?[iI]nflict

[Ss]elf[- ]?[iI]mmolat

[Cc]utt

[Hh]ead[- ]?[Bb]ang

[Oo]verdos

[Hh]opelessness

[Pp]owerlessness

[Hh]elplessness

[Nn]egative[- ]?[Aa]ttitude

[Ee]motional[- ]?[Nn]egativism

[Pp]essimism

[Dd]epress

[Pp]assivity

[Ss]ad[- ]?[Aa]ffect

[Ss]adness

[Dd]ecreased[- ]?[Aa]ffect

[Cc]ognitive[- ]?[Rr]igidity

[Ss]uicidality

[Ss]uicide[- ]?[Ii]deation

[Mm]ental[ -]?[Hh]ealth

[Mm]ental[ -]?[Hh]ealth[ -]?([Cc]ris[ei]s|emergenc)

([pP]sychiatric|[Pp]sychotic|[Ss]chizophren\w*|[Bb]ipolar|[Mm]ental\w* ([Ii]ll\w*|[Dd]isorder))[ -]?([Cc]ris[ie]s|[Ee]mergenc|[Aa]cute)

([Cc]ris[ie]s|[Ee]mergenc\w*|[Aa]cute)[ -]?([pP]sychiatric|[Pp]sychotic|[Ss]chizophren\w*|[Bb]ipolar|[Mm]ental\w* ([Ii]ll\w*|[Dd]isorder))

**Summary of quality assessment criteria for reasonable quality studies**

| **Study design** | **Assessment tool** | **Questions from the overall scale number** | **Criteria to be met** |
| --- | --- | --- | --- |
| Cohort study | JBI | 1 | Two groups were similar and recruited from the same population |
|  |  | 2 | Exposures were measured similarly to assign exposure status |
|  |  | 6 | Were strategies to deal with confounding factors stated |
|  |  | 7 | Suicidal behaviour was measured in a valid and reliable way |
|  |  | 9 | Follow-up was complete or reasons for loss to follow up were described and explored |
| Before and After | NIH tool | 3 | All eligible participants that met the prespecified entry criteria were enrolled |
|  |  | 6 | Outcome measures were prespecified, clearly defined, valid, reliable, and assessed consistently across all study participants |
|  |  | 7 | The same approach/data source was used for the pre-covid measures of suicidal behaviour as those collected during the pandemic period |
|  |  | 8 | There were no differences in level of missing data pre and during the pandemic period |
| Time series | EPOC RoB Tool | 1 | Intervention independent of other changes |
|  |  | 2 | Intervention unlikely to affect data collection (low or unclear risk) |
|  |  | 5 | Missing outcome measures were unlikely to bias the results |
| Cross sectional | JBI | 1 | Criteria for inclusion was clearly defined |
|  |  | 2 | Study participants and setting were described in detail |
|  |  | 6 | Were strategies to deal with confounding factors stated |
|  |  | 7 | Suicidal behaviour was measured in a valid and reliable way |
| Case series | JBI | 1 | Criteria for inclusion was clearly defined |
|  |  | 3 | Valid methods were used for identification of the condition for all participants included in the case series |
|  |  | 4 | Consecutive cases were included |
|  |  | 6 | Clear reporting of demographics of the participants in the study |

JBI – Joanna Briggs Institute; NIH – National Institutes of Health; EPOC – (Cochrane) Effective Practice and Organisation of Care; RoB – risk of bias

| Author (year) | Number of cases* by pandemic period | |
| --- | --- | --- |
|  | Pre- | During |
| Thongchuam (2021) | 1 | 7 |
| Fidanci (2021) | 187 | 31 |
| Eray (2021) | 23 | 11 |
| Acharya (2020) | 9 | 4 |
| Sengupta (2020) | 33 | 50 |
| Behara (2021) | 105 | 61 |
| Jhanwar (2020) | 3 | 11 |
| Shrestha (2020) | 38 | 55 |

**Number of cases of suicide and self-harm used to calculate rate ratios**

* Cases were either suicide deaths or self-harm attempts
