# Supplementary material for: Suicide and self-harm in low- and middle- income countries during the COVID-19 pandemic: A systematic review
Source: PLOS Glob Public Health. 2022 Jun 1;2(6):e0000282. doi: 10.1371/journal.pgph.0000282 (PMC10021274; doi:10.1371/journal.pgph.0000282)
Supplement: Attachment — Submitted filename: responsetoreviewer_blank.docx [file pgph.0000282.s005.docx]

There were no reviewer comments to address
